# A Drugged Swimmer

**Published:** 1875-09

**Authors:** 


### A DRUGGED SWIMMER.

On the twenty-second day of July, 1875, one Thomas Coyle of Chester, Penn., and J. B. Johnson of England, undertook to swim against each other, a distance of some thirteen miles in the Delaware river, for the sum of $2,000. Both were rapid swimmers and both had often exhibited great powers of endurance. Johnson had often swam for money, and had repeatedly distanced able competitors. Coyle had astonished close observers by his success, and entered upon the race with the inward certainty of triumph. Both men had been carefully exercised and appropriately fed, in the opinion of their trainers, and both were pronounced in capital physical condition.

In forty-seven minutes from the moment of starting, Coyle had swam three miles and Johnson was fully three-quarters of a mile behind him. Proverbially, "a stern chase is a long one," and it is reasonable to conclude that Johnson would have found it difficult to overtake his active antagonist, had not that antagonist ignorantly committed an act which lost him the race, and might easily have destroyed his life.

Both men were attended by friends in boats. It was supposed to be for the interest of these attendants to sustain and encourage their respective clients. So far as we have heard, Johnson's attendant did his duty by his man. But Coyle's friend foolishly poisoned Coyle, and so lost the race for him.

This was the way the poisoning was accomplished, according to the papers. Elated with his promise of success, he took a heavy draught of a mixture which had been prepared for him by a "Doctor," which mixture the friend in the boat handed to the swimmer. Coyle did not pretend he was fatigued; he did not claim that he needed anything. He declared that he felt all right, swallowed his enormous draught, and again pushed out.

The poison which Coyle had swallowed did not instantly exhibit its peculiar power. The advantage in the race remained with him for some time. Perhaps, in spite of the potion, he would have won, though the chances were against him from the instant that he swallowed the doctor's dose. But Coyle was not content with his single draught, excessive though it was. When he still had five miles to swim and Johnson was yet far behind, he bethought him that he would take another dose. This was permitted by the attendant, and two minutes thereafter the race was practically ended. The swimmer, so strong and so successful naturally, was fairly conquered by the potent poison. His movement ceased and he sank, powerless even to sustain his body in the water. He was taken on board the boat; medical aid was secured and after several hours of unconsciousness, he was pronounced out of danger. His competitor in the race leisurely swam the entire distance and came out an easy winner, fresh to the last.

Coyle said he was poisoned. All those who had foolishly staked money on the chance of his success, said so too.

Well, they were right. Coyle was unquestionably poisoned, drugged. The drug which he swallowed was one of the most potent poisons known to the chemical art. It never yet failed to kill, if properly taken. It has been used thousands of years, and always with success. It would have killed Coyle but for the friendly boat near by, and the skill and care which followed. Had the dose been a little larger, or once again repeated, no skill, however great, could have saved him. But, luckily, he escaped.

We said "luckily," but probably we were in error. If this athlete could but learn the real lesson which his failure teaches, his escape from death might be deemed fortunate. But he will not, we fear. He sees the same poison used every day, and people live through it. He will continue to swallow it and will surely fail just as often as he takes the dose.

The poison was *whiskey*. The "doctor" who prepared it is reported to have mixed ginger with it—tincture of ginger, probably—which was adding more and stronger alcohol to that of the original poison. Alone, the ginger would have done no special harm, though it would have been far better if even this warming substance could have been kept till the race was ended.

The "doctor" committed a grievous error if he advised the use of this poisonous mixture under the circumstances. While the body is exposed for a long time to a temperature below the natural blood-heat, all the powers of nature are taxed to keep the body warm. Nature is able to do this for a long time if she is let alone. But if you add to her work by opposing to it any drug the tendency of which is to lessen the vital heat, you give her more work than she can do, and she gives up the effort. This is just what Coyle did. He swallowed two heavy doses of a drug, which more absolutely than any other known, reduces animal heat. To lower the natural heat considerably is dangerous and often fatal. The standard is 98° Fahrenheit; to reduce it to 96° may destroy life. No human being ever yet drank whiskey copiously, without reducing the temperature of the blood to some extent. To drink it when exposed to cold, is doubly dangerous, because the poison and the cold work together tc produce the same fatal result.

Coyle was poisoned; had he taken strychnine, this fact would not have been more apparent. The lesson to be learned here is, simply, that of all things to be avoided when important tasks—bodily or mental—are to be performed, nothing should be more carefully shunned than alcohol. When all the doctors understand, this and substitute some less potent agent, the world will be better off.

